# Acceptance and commitment coaching for music performance anxiety in adolescent singers

**DOI:** 10.3389/fpsyg.2024.1386559

**Published:** 2024-08-20

**Authors:** Anupa Elizabeth Paul, David G. Juncos, Debbie Winter

**Affiliations:** ^1^Independent Researcher, Esslingen, Germany; ^2^Voice Study Centre, University of Wales Trinity St David, East Bergholt, United Kingdom; ^3^Independent Researcher, Philadelphia, PA, United States; ^4^Voice Study Centre, Associate College of Essex University and Collaborative Partner of University of Wales Trinity St David, East Bergholt, United Kingdom

**Keywords:** adolescent singers, puberty, performance anxiety, performance enhancement, flow

## Abstract

**Introduction:**

Most of the anxiety disorders, particularly social anxiety, seem to develop either during childhood or adolescence. Adolescent singers who experience physical, mental and emotional changes along with voice change are particularly prone to the development of MPA. However, adolescence also seems to be an opportune time to instil healthy behaviours in singing students as they are more likely to remember these coping strategies, owing to the release of dopamine and the ‘reminiscence bump’. As this period of a singer’s life is wrought with inevitable anxiety development, the additional aim of the study was to develop a coaching framework which can be used by singing teachers in their practice. Mindset training for adolescents seems crucial to help them continue singing through puberty so they do not drop out of singing lessons or choir during voice change and identity development.

**Methods:**

The study aimed to determine if Acceptance and Commitment coaching could be used with adolescent singers with MPA and to record and analyse rich qualitative data in the form of semi-structured participant interviews and questionnaires.

**Results:**

When their perception of MPA symptoms and physiological and psychological arousal before a performance changed, their behaviour and reactions changed accordingly. The change took place over a period of time, which was characterised by discoveries about themselves, confusion in understanding new concepts and letting go of old habits. Interestingly, by the end of the coaching sessions, their preoccupation with pleasing the audience and appearing perfect on stage was replaced by a new-found delight in pursuing values and goals related to their singing. Along with this came the acceptance of themselves and others as individuals with the potential for growth and change and the capacity to learn from mistakes.

**Discussion:**

This study marks the first investigation into the effectiveness of using of ACC as an intervention for MPA in adolescent singers undergoing puberty by a singing teacher. The results are promising and suggest that ACC is an effective MPA intervention for adolescent singers to cope with inevitable development-related anxieties and keep them engaged in the activity of singing during their pubertal years.

## 1 Introduction

Puberty in adolescence represents a period of physical upheaval and transformation (Sweet, 2015). The physical and emotional health of the singer is inextricably connected and the pedagogy of the adolescent singing voice needs to consider several aspects for the healthy development of the singer (Gebhardt, 2016). Music Performance Anxiety, with its symptoms of persisting, distressful apprehension, has been seen to be widespread among adult singers (Kenny, 2011; Patston and Osborne, 2015). Although MPA has been noticed in children, its origins have not been studied in detail (Patston and Osborne, 2015). While it is expected that students would become more comfortable with performing with an increase in expertise and familiarity with teaching methods, research shows that MPA increases throughout adolescence, peaking at the age of 15 years (Patston and Osborne, 2015). The average age of onset has been observed to be falling worldwide, with the current age of onset around 12.5 years for boys and 10 years for girls, compared to 14 years for boys and 12 for girls in 1950 (Andrews and Summers, 2002; Gackle, 2014; Williams, 2019). Pubertal timing or going through puberty earlier, later or at the same time as one’s peers seems to have an effect on the development of psychopathology, which may well persist into adulthood (Graber, 2013). Early-maturing girls and late-maturing boys may be more prone to developing depressive disorders and social anxiety (Graber, 2013). Males who begin voice change earlier than their peers may not perceive themselves as good singers and avoid participating in middle school choir (Fisher, 2014). Although the changes observed in girls are gradual in comparison to the drastic and tumultuous changes observed in the male larynx, female adolescents show characteristic changes such as breathiness, huskiness, insecurity of pitch, decreased range and voice cracking (Gackle, 2014; Sweet, 2015). Young female singers reported a loss of vocal confidence with the onset of voice change, and this was usually accompanied by a loss of confidence in other areas of their lives as well (Gackle, 2014).

Apart from the changes in the body related to puberty, the adolescent brain is also changing during this time and continues to do so into the twenties (Mills and Anandakumar, 2020). These brain changes manifest in behavioral changes such as the desire to explore and form new friendships and social bonds (Mills and Anandakumar, 2020). Development of an organized and coherent sense of identity or a sense of self is considered as the key task in adolescence (Crocetti et al., 2008). Musical identity development in children is influenced by the family, predominantly the parent’s beliefs about the child’s musical talent and how they regard the child as a musician (Davidson and Borthwick, 2002). Adolescents who are highly invested in their musical identities might find it difficult to separate their self-esteem from their musical self-efficacy (Kenny, 2011). Such adolescent singers may be more prone to anxiety because failure as singers could be interpreted as failure as people (Chesky and Hipple, 1997). The fear of failure, which has its origins in childhood, could be due to high parental expectations, where the child defines failure as unacceptable and have negative implications for their self-worth and relational security (McGregor and Elliot, 2005). Thus, the child seeks to actively avoid achievement situations such as sports or music as they are vigilantly trying to avoid failure. For adolescents high in fear of failure, achievement situations such as music performance are not opportunities for improving but could be threatening and potentially shameful, thus creating a self-perpetuating cycle which maintains and exacerbates this fear (McGregor and Elliot, 2005). With regard to music learning, adolescents may be frustrated by motor skills which take time to develop and may lack the motivation to practice and perform due to fear of failure (McGregor and Elliot, 2005). This situation could be exacerbated by the fact that they are also learning to adjust the motor coordination of their rapidly growing bodies (McGregor and Elliot, 2005). However, if they avoid practice and performance opportunities, they miss out on important parts of their learning.

The adolescent brain also gains heightened sensitivity to mundane events because of the increased release of dopamine during this period of growth (Gebhardt, 2016). When released with strong emotions such as shame or fear, dopamine makes it possible for ordinary events to become engrained in the memory (Gebhardt, 2016). Psychologists refer to this as a ‘reminiscence bump’ as it involves the ability to recall experiences between the ages of 10 and 18 more clearly than any other period of life (Gebhardt, 2016). A majority of adult musicians in a study indicated that their first memories of music performance anxiety were from adolescence, and they could recall these memories with great detail as if they had happened just the day before (Kenny and Osborne, 2006). In the case of adolescent musicians, many of whom are forced into performance situations early in life owing to their giftedness in musical development, anxiety responses can lay down templates for subsequent reactions to music performance situations throughout life (Kenny, 2011). Thus it is evident that embarrassing or shameful performance-related situations in childhood and adolescence may have a way of staying deeply ingrained in a person’s memory and may potentially guide behavior and career choices in the future.

Young adolescents also show an increased sensitivity to evaluation or criticism in this stage of life (Slee, 1993; Hoxter, 2017). This is due to a change in their thinking and reasoning abilities, where the teenager has an “imaginary audience” constantly monitoring and assessing them (Slee, 1993; Kenny, 2011). This leads to heightened self-consciousness and peer-group conformity, with the changes happening to them taking center stage in their personal thoughts (Kenny, 2011). They also believe that others are concerned about their appearance and behavior as much as they are (Slee, 1993; Vartanian, 2000; Kenny, 2011). It seems that adolescents who may be prone to social anxiety could develop music performance anxiety co-morbidly along with maladaptive perfectionism (Dobos et al., 2019). Female high school musicians reported higher performance anxiety symptoms, corresponding with research showing higher shame and social anxiety symptoms in this gender in adolescence (LeBlanc et al., 1997; Ranta et al., 2007; Williams, 2019). MPA and Perfectionism also has a steeper and more intense developmental trajectory in females than males (Patston and Osborne, 2015). While social-phobics would rather avoid social situations, musicians with social phobia need to perform in public in order to excel and this could in turn lead to intrapersonal tension (Dobos et al., 2019).

Music performance anxiety develops as a result of genetic predisposition and the individual’s learning history (Barlow et al., 2004; Mineka and Zinbarg, 2015; Patston and Osborne, 2015). Teachers who provide positive and supportive guidance to children and adolescents during singing lessons and performance situations, choosing repertoire that is appropriate to the developmental stage of the adolescent are more likely to contribute to a positive learning history (Osborne and Kenny, 2005). The style of singing lessons and the temperament of the singing teacher are expected to affect the young student’s enjoyment of singing and also their learning outcomes (Patston and Osborne, 2015). Teachers are also seen to contribute to the development of perfectionism and anxiety in their students if they themselves have these tendencies and unwittingly pass them on to their students (Patston and Osborne, 2015). Additionally, research shows that musicians suffering from MPA are more likely to seek help from their teachers rather than a clinical therapist (Williamon and Thompson, 2006). While it seems that adolescents were prone to the development of social anxiety and MPA, it also seems possible that this might provide a unique window of opportunity to instill healthy behaviors and coping strategies in singing students (Gebhardt, 2016). It might be crucial for the singing teacher to provide interventions during this period of life alongside singing lessons to avoid the development of maladaptive coping strategies.

As most of the research on MPA interventions has been undertaken in clinical settings, there is very little teacher-led coaching research done for adolescents (Shaw et al., 2020). The present study aims to research and deliver a coaching course for adolescent singing students dealing with MPA in the private voice studio setting, while addressing the specific developmental needs of teenage singers (Patston and Osborne, 2015).

## 2 Acceptance and commitment coaching as a treatment for MPA in adolescent singers

One of the strategies gaining popularity in clinical and coaching settings is Acceptance and Commitment Therapy (ACT), a newer third-wave intervention framework (Eifert and Forsyth, 2005; Juncos and de Paiva Pona, 2018; Hill and Oliver, 2019). ACT is based on the philosophy that thoughts and emotions need not be managed in order to live a valued and meaningful life (Eifert and Forsyth, 2005). Instead of controlling our internal private world, ACT focuses on accepting unwanted thoughts and feelings, whose occurrence and disappearance cannot be controlled, and on commitment and action toward living a.

valued life (Eifert and Forsyth, 2005). In the case of Music Performance Anxiety (MPA), ACT suggests that anxiety need not stand in the way of doing what one loves, in this case, music performance (Eifert and Forsyth, 2005; Juncos et al., 2017). Experiential avoidance, or the individual’s attempts and efforts to avoid, suppress or alter negatively perceived body sensations, thoughts, worries and memories, is considered to be at the core of all anxiety disorders (Eifert and Forsyth, 2005). ACT principles aim to allow the individual to overcome rigid and inflexible patterns of experiential avoidance by allowing them to approach fear and anxiety more fundamentally and deeply (Eifert and Forsyth, 2005).

The ACT Hexaflex Model is a visual representation of the six core processes of Acceptance and Commitment Therapy (ACT). The six core processes are.

1. Acceptance (the willingness to experience difficult thoughts and emotions).
2. Cognitive Defusion (Separating ourselves from our thoughts and emotions).
3. Being Present (Engaging fully with the present moment).
4. Self as Context (Recognizing that we are not just a product of our thoughts and feelings).
5. Values (Beliefs and principles that guide our behaviors).
6. Committed Action (Choosing to engage in behaviors which align with our values).

The core processes are interconnected and mutually reinforcing and contribute to the development of psychological flexibility in the coachee.

ACT has been used with adolescents in clinical and school settings in areas such as chronic pain, chronic fatigue, depression, stress, obesity and anxiety (Livheim et al., 2014; Kemani et al., 2018; Kallesøe et al., 2020; Zetterqvist et al., 2020; Clery et al., 2021; Guerrini Usubini et al., 2022; Petersen et al., 2022) The results of these studies have been promising, with participants showing a significant decrease in psychological inflexibility and also in symptoms (Livheim et al., 2014; Kemani et al., 2018; Kallesøe et al., 2020; Zetterqvist et al., 2020; Clery et al., 2021; Guerrini Usubini et al., 2022; Petersen et al., 2022). Online interventions in group settings have also been developed in order to increase the accessibility of ACT among adolescents, with results showing benefits such as symptom reduction in clinically diagnosed youth (Puolakanaho et al., 2018; Keinonen et al., 2021; Lappalainen et al., 2021). ACT has been used in MPA studies in university music students with clinically significant improvements in psychological flexibility along with symptom reduction (Juncos and Markman, 2015). Although used so far in clinical settings and administered by performance psychologists, ACT has been recommended to be used as an evidence-based model of care by music teachers and practitioners over the other available options (Juncos and De Paiva Pona, 2018). ACT is also seen to be a promising intervention for vocal students affected by physical MPA symptoms (Juncos et al., 2017).

Acceptance and Commitment Coaching (ACC) is a model of coaching based on the principles of Acceptance and Commitment Therapy (ACT) (Hill and Oliver, 2019). ACT, Mostly used in clinical settings for a range of issues like psychosis, depression, anxiety, addiction, and chronic pain, has an extensive body of research demonstrating its effectiveness in helping people make changes in their lives (Hill and Oliver, 2019). Due to this evidence base and versatility, ACT has been adopted by coaches in several settings like sports, music and the workplace (Bond et al., 2008; Blonna, 2010; Skews, 2018; Hill and Oliver, 2019). In one study, ACC appears to be a promising MPA intervention that can be administered by a music teacher in non-clinical settings (Shaw et al., 2020). Therefore, ACC could be an effective intervention that music teachers can use for adolescents experiencing MPA in individual and group settings, either through online or in-person sessions.

### 2.1 Aims of this study

This study aimed to be the first application of ACC in which a singing teacher administered the interventions directly to individual adolescent singers undergoing puberty and experiencing MPA. The research question to be answered was: Can ACC be used as an effective intervention for adolescent singers with MPA by a singing teacher who has no training in psychotherapy? It was hypothesized that there would be a significant improvement in the singer’s psychological flexibility after receiving 6 sessions each with the singing teacher. In addition, it was hypothesized that ACC could improve the experience of flow during practice and performance, which would encourage the singers to keep returning to the activity of singing during their pubertal developmental years.

The study’s main aim was to determine the changes in the participant’s self-perception of MPA symptoms and whether the ACC intervention could motivate the adolescent singer to continue pursuing the activity of singing during the pubertal years. No attempt was made to interfere with or record the change in quality of the actual singing performances before and after the intervention.

## 3 Materials and methods

### 3.1 Participants

The participants were 4 singers (3 male and 1 female) between the ages of 13 and 17 years. They were recruited through word-of-mouth advertising through the researchers’ teaching contacts. None of the singers were the researcher’s singing students. The participants filled out the KMPAI-A questionnaire in order to ascertain if they had MPA and were hence eligible to participate in the study.

### 3.2 Coach

AP is an independent voice coach and choral trainer. She was trained as a western classical singer and has performed as a soloist and along with renowned choirs both in her hometown Chennai, India and internationally. She became interested in the topic of MPA after she noticed the occurrence of MPA in her singing students and wished to find effective evidence-based strategies which could be used in the teaching studio in tandem with singing lessons. As part of her M.A. in Voice Pedagogy, she conducted an in-depth literature review on the effects of voice change and puberty on the development of MPA in adolescent singers. She concluded that adolescence would be a unique window of opportunity to train young musicians to cope with inevitable developmental anxieties.

Institutional review board (IRB) for this research was given by the University of Wales Trinity St David’s (UWTSD) ethics board. AP conducted this research with the Voice Study Center, Ltd., a provider of postgraduate voice pedagogy study that is affiliated with the UWTSD. AP was supervised by the third author (DW) in securing ethics board approval and in ensuring that AP provided the intervention as a coach and not as a psychotherapist to the participants.

### 3.3 Procedure

#### 3.3.1 Training

AP received training in ACC from the second author (DJ), a clinical and performance psychologist with more than 15 years’ experience in treating anxiety disorders and specific expertise in using ACT with musicians to enhance performance and treat MPA. Materials for AP’s training and the participant’s coaching were taken from three books: Acceptance and Commitment Training for Musicians by Juncos and de Paiva Pona (2018), Acceptance and Commitment Coaching by Hill and Oliver (2019) and ACT for Treating Children by Tamar Black (2022).

#### 3.3.2 Coaching intervention

AP met each participant individually over Zoom for six sessions between March and June 2022. The sessions ranged between 45 to 60 min each depending on the participant. The participants performed regularly either as vocalists with bands or in choirs or in karoake and stage shows. The two older participants, both 17 years old at the time, enjoyed the mindfulness exercises which were either ‘eyes-closed’ or body-scan observations. However, the two younger participants (13 and 14 years) required mindfulness exercises that were activity-based. There were no fixed coaching plans for all the participants, rather the ACT Kidflex based on Black’s book was used to explain concepts of acceptance and commitment to performance values and to develop psychological flexibility in the participants. It is also important to note that not all participants required training in all the 6 core processes.

As this study was focused on delivering coaching interventions to individuals, AP had to take into account the age and personality of the participants while deciding the appropriate exercises and metaphors for the participants. Participant A, a 17-year-old male, was able to understand abstract ideas and metaphors well. He was also seen to ask questions and reflect and process thoughts verbally. He was the only participant who liked the eyes-closed or focus-based mindfulness exercises. AP began with the core processes of Being Present followed by Cognitive Defusion, Values and Committed Action. Participant B, a 14-year-old male, did not enjoy discussing his thoughts and feelings verbally. He also did not enjoy eyes-closed mindfulness exercises. However, AP found him deeply engaged in written/painting activity-based mindfulness exercises, with some of his finished work being elaborate and descriptive of his MPA-related thoughts and symptoms. AP also found it easy to connect the ACT Hexaflex principles to his current superhero character. AP began with the core process of Self as Context followed by Acceptance, Being Present, Cognitive Defusion and Committed Action. Participant C, a 13-year-old male, did not always have the appropriate words to describe his feelings. With this participant, AP found it challenging to understand if he had grasped the Hexaflex concepts. As he was not yet able to understand abstract concepts, AP used exercises meant for children, which he then responded to very well. The core processes used with him were Being Present, Values and Committed Action. Participant D, a 17-year-old female, struggled with shifting her attention away from her MPA symptoms, which included extreme tremors in her hands and voice when she went up to sing and accompany herself on the piano. AP began with values-related exercises with her, rather than mindfulness exercises. The core processes used with this participant were Values, Committed Action, Self as context and Cognitive Defusion.

As a common theme, all the participants were primarily preoccupied with how their peers and audience viewed them and their performances. During the initial sessions they found it difficult to understand the concepts of acceptance of anxiety symptoms as they felt they needed to get rid of the symptoms in order to have enjoyable performances. By the end of the sessions they came to accept the symptoms as something normal and as part of their performances.

The participants completed questionnaires and gave semi-structured interviews both before and after the coaching intervention sessions.

The questions which were asked in the pre-coaching questionnaire were

1. What are the symptoms or feelings that you are aware of before of while you sing?
2. Do these symptoms/feelings interfere with your singing?
3. Have you ever tried to make these symptoms go away? How?
4. What is it about singing for an audience (of any number) that scares you the most?
5. What would your ideal performance feel like?

Questions included in the post-coaching questionnaire were

1. What have you learnt during the coaching sessions?
2. How are you going to use what you have learnt?
3. What have you learnt about yourself during the coaching sessions?
4. What is your current outlook on singing performance? Has it changed or remained the same after the coaching sessions?
5. What are you most looking forward to as a singer and performer?

### 3.4 Semi-structured interview questions

#### 3.4.1 Pre-coaching

1. Before you go up to sing or while you perform, what kind of symptoms do you experience?
2. While you are singing what do you often think about?
3. What does nervousness feel like in your body?
4. How long before the performance do these sensations appear? Days? Hours? Minutes before?
5. Does the intensity of these sensations change during the performance?
6. How do you practice or prepare for an upcoming performance?
7. Describe a performance that went really well
8. Describe a performance that did not go so well, if any.

#### 3.4.2 Post-coaching

1. What was the most helpful/useful part of the coaching sessions for you?
2. What was the least helpful/useful part of the coaching sessions for you?
3. How do you feel it went over Zoom rather than in-person?
4. What do you feel about the length of the course and the duration of each session?
5. Do you feel your peers and other performers talk about MPA? Would you talk about your MPA with them? Why or why not?
6. Is there anything in the sessions that you struggled with? Anything you found difficult to understand or wished we had spent more time on?
7. Would you recommend these sessions to other singers you know who may or may not be struggling with MPA?
8. Do you feel equipped to continue using the exercises and principles which you learnt during the sessions?
9. What do you think of the exercises? Were they fun/difficult/boring/interesting?
10. Is there any way that the content of the coaching sessions could be improved?
11. What did you learn about mindfulness/acceptance of MPA symptoms and pursuing valued actions over the last few weeks?

Exercises used for different ACT processes used during the coaching sessions:

(The exercises were taken from ‘ACT for Musicians by David Juncos and Elvire de Paiva e Pona and ACT for Treating Children by Tamar Black)

#### 3.4.3 Being present

1. Scanning a Picture frame with your eyes open
2. Mindfulness of the body

#### 3.4.4 Acceptance

1. Tug of war with anxiety monster
2. The Willingness Dial
3. The Pen Exercise

#### 3.4.5 Cognitive defusion

1. Thought labeling
2. Word Repetition to form new associations
3. Thanking your mind for your thoughts

#### 3.4.6 Self as context

1. Clouds in the sky
2. Chessboard

#### 3.4.7 Values

1. Heart exercise
2. Two roads
3. Writing mission statements

#### 3.4.8 Committed action

1. Specifying Performance-related goals

## 4 Findings

AP set out to conduct this study in order to ascertain whether ACC would be a feasible intervention which can be used by private singing teachers with their adolescent singing students who experience MPA. AP also wished to understand the experiences of teenagers who have MPA. Adolescents, due to natural growth processes are prone to the development of MPA along with or independent from social anxiety (Kenny, 2011). Since this is the beginning stage of MPA, which is then maintained through to adulthood, it was necessary to find an intervention which singing teachers, who are not trained in psychotherapy, can use in their private teaching practice.

The findings revealed a definite change in the participants and that change began with their way of thinking about MPA. When their perception of MPA symptoms, physiological and psychological arousal before a performance changed, their behavior and their reactions changed accordingly. The change took place over a period of time which was characterized by new discoveries about themselves, confusion in understanding new concepts and letting go of old habits. Interestingly, by the end of the coaching sessions their preoccupation with pleasing the audience and appearing perfect on stage was replaced by a new-found delight in pursuing values and goals related to their singing. Along with this came the acceptance of themselves and others as individuals with potential for growth and change and the capacity to learn from mistakes.

The following themes emerged.

## 5 Theme one: we’re going on a roadtrip!

### 5.1 The starting point

#### 5.1.1 Going in circles

As in every journey that is undertaken, unless there is clarity on where the traveler intends to go, there is a lot of time and energy wasted on going in circles and stopping at or passing through unplanned destinations. Before the start of the coaching sessions the participants had a vague idea about what they wanted from a good performance. They depended more on feelings to tell them if the performance had gone well. There was a common idea that a good performance would also feel good. However, when they encountered feelings of discomfort from singing in front of people and because they depended on good feelings to evaluate their performance, they either shamed themselves for feeling that way or tried different strategies to get rid of the discomfort before moving on in their singing journey. But, the more they tried the strategies, the more frustrated they became. The older two participants seemed especially confused that they were not able to get rid of this discomfort and other nervous symptoms, despite their singing experience. The participants ultimately decided that the best solution for their MPA was more practice before a performance, as this might make them feel more confident before they went on stage. But they could never decide how much practice was enough.

They all had had at least one good performance in the past, when they had experienced a good feeling before they had gone on stage. They had practiced and rehearsed before those performances and hence had concluded that in order to produce the same good feelings before a performance, long practice sessions had to be the key to unlock these good feelings. However, during interviews the participants also confessed to not practicing at all or practicing only if there was a performance coming up in the near future. This contradicted with their beliefs regarding practice affecting performance.

Some of the participant responses are shown below. The core process which may need to be trained has been indicated in brackets.

Participant A (PA) - (Self as context, Cognitive Defusion)

“If there’s like a good feeling”

“Be very, very well prepared”

“I thought the more I performed, it will get lesser. It’s not happening”

Participant B (PB) - (Cognitive Defusion)

“Feel proud of myself”

“Satisfaction which I feel”

Participant C (PC) - (Values)

“How I feel at the end”

“The applause”

“If I mess up what’s gonna happen?”

Participant D (PD) (Self as Context, Cognitive Defusion, Acceptance)

“Should feel very relaxed”

“Should have no nervousness or goosebumps”

“Still takes place each time”

#### 5.1.2 Audience

The participant’s journey was also overly dependent on the real and imaginary audience. The participants felt they were moving forward only if the audience approved their performance. Even while preparing for their performance, the imaginary audience was always consulted with regard to repertoire selection. The participants also tried to modify their behavior based on how they felt the audience was reacting to them. Irrespective of the number of the audience, the presence of known or unknown people triggered varying reactions in the participants. Audience opinion was very important just before, during and even after the performance had ended, with participants actively scanning the audience’s facial reactions while singing. The level of audience applause was deemed as the feedback needed to assess if the performance had gone well or not. The participants seemed to vacillate between depending on good feelings and audience approval, making performance evaluations entirely on these criteria, thereby ignoring their singing values.

PA (Cognitive Defusion)

“Kind of avoid making eye contact”

“Appeal to the audience”

“We will look at how the audience reacts in like, the first 30 seconds”

PB (Being present)

“Scared that the audience will lose interest”

“What they are looking for”

PC (Self as context)

“It’s like a weight on my mind”

“Audience plays a very important part”

PD (Being present, Cognitive defusion)

“Will everyone like it?”

“The energy was different. They were hard to engage. I panicked”

### 5.2 The crisis/turning point

During the initial sessions, the participants seemed resistant to the ACT principles. Instead they wished to learn quick fixes to get rid of their uncomfortable feelings. When they began to understand the point of the exercises, they essentially got over their boredom in doing them. But this took between three to five sessions, depending on the participant. As they were given choices between different exercises they ended up choosing the ones they liked best to practice between sessions. They also pressed through confusing and sometimes very different ways of thinking about their MPA symptoms, anxiety triggers and their reactions.

The first signs of change were seen after 2–3 sessions. The ACT core processes which were being trained have been indicated in brackets.

PA (Acceptance)

“So the aim of these exercises are not for it to be fun or something you enjoy or anything. It is work. It’s to build your focus and attention and all that. So yeah …”

“Even though you can practice as much as you want, you can be as confident as you want. There are certain things you can’t account for on the day of the performance. You are bound to get nervous and all that and that is fine.”

PB (Committed action)

“I kind of got over the boring feeling because I realized that it was necessary; it was helpful. And so I will admit that when I do the exercises, I do get a little bored. But I kind of think about how it’s benefitting me.”

“After the first two sessions, I think I was a little bit confused, like what we had been talking about, but then eventually, they kind of got a little more in detail. And ironically I was able to understand it better.”

PC (Acceptance)

““I found that staring into one thing was difficult for me. Apart from that everything was interesting. Yes”

“I learned that I can’t just get rid of negative thoughts. I just have to change the way of thinking.”

PD (Cognitive defusion, acceptance)

“So it definitely.it is helpful. But it was slightly boring. But I found that noticing the thoughts when they passed by you. I found that interesting.”

“We have to live with anxiety. That is one of the most key points I learned”

Once the participants learnt to face their feelings and accept them, values were clarified and they were given a chance to indulge in metacognition, or the act of thinking about their thinking. They recognized what they were used to believing about themselves and their performance. Toward the last few sessions, they learned to defuse from beliefs that did not serve them and focus on values-related beliefs instead. They had the opportunity to re-affirm their values related to singing.

PA (Values)

“I subconsciously avoid uncomfortable situations such as singing in front of large audiences. I’ve learnt that I’m a good singer and performer, and there is no need to feel any different.”

PB (Self as context)

“I can be very quick to judge myself and it can often be demotivating. I have learnt that it is necessary to be firm but not harsh as well”

PC (Committed action)

“I’m not such a shy person actually and can achieve my goals. I will appreciate everything I am and will be “

PD (Cognitive defusion, self as context)

“I have learnt that the way I think changes my performance”

### 5.3 The journey continues

Clarifying values in turn gave the participants a clear picture on where they intended to go. The shift was seen from dependence on feelings and audience approval to focusing on steps to take toward valued behavior. They were also able to assess how far they had strayed from the path toward their destination. Different exercises helped them assess and visualize where they were currently. Course correction was necessary. Participants also realized that the journey they had undertaken was like a one way journey facing due north. Milestones had to be acknowledged and celebrated along the way but the journey was always ongoing. Rather than looking at performances as potentials for rejection and criticism, they were beginning to be viewed as opportunities to have fun while pursuing valued behavior. More importantly, mistakes which were earlier considered unacceptable and shameful became potential areas for future improvement.

PA (Values, committed action)

“Looking forward to playing outside my comfort zones. I want to keep singing and performingwith my band and just enjoying playing with them. Seeing myself grow as a singer and performer”

“I want to keep singing”

“I subconsciously avoid uncomfortable situations”

“These sessions have like helped to identify areas where I may not be as confident”

PB (Values, acceptance)

“Looking forward to be a better singer and performer”

“learn from mistakes”

“I’m very quick to judge myself”

“I think that I was constantly trying to like bottle up the emotions”

“Your mind is like a muscle, you have to train it..like, it constantly needs like substance.”

PC (Acceptance, values)

“I will happily accept whatever comes”

“train the mind”

“I am hoarding unnecessary anxiety”

“I realized how far I was from my goal. Now I am a little more closer.”

“Singing is not just a showcase of your talents. It’s an adventure.”

PD (Values, committed action)

“Get to a level where I can sing with maximum confidence”

“learning about me”

“I’m not so far from my goal. I have been accomplishing things”

“If there is no mistake then it will be boring. There won’t be anything to accomplish in the next performance”

### 5.4 Theme two – how the journey changed me

#### 5.4.1 Accepting myself

Participants realized that the journey changed them in several ways. The highlight of this journey was that they no longer saw anxiety and its symptoms as something strange but as an aspect to be expected when something of value is attempted by them. Mistakes in performance previously seen as an extension of themselves as people were no longer feared but seen as opportunities to improve in future performances. All the participants exhibited the core process of Acceptance.

PA

“It is normal to feel nervous before or during a performance”

PB

“Making mistakes or feeling nervous is normal”

PC

“Anxiety can be faced”

PD

“Anxiety is part of being a singer”

#### 5.4.2 Accepting others

Along with accepting themselves, participants also empathized with other singers and realized the futility of setting impossible standards and being critical of themselves and others. There was a shift from viewing fellow performers as just singers. They are now viewed as humans with flaws and imperfections who sing and create art. Participants also understood why their peers refused to talk about or acknowledge MPA because they had been that way before the coaching sessions. They understood that talking about it and accepting that one might have uncomfortable feelings associated with singing might ruin their social image and reputation.

Singing requires the singer to be vulnerable in front of the audience, causing adolescents, especially males, to choose to play instruments and stay at the back of the stage but still be part of the performance. This is pertinent as singers who face MPA are different to instrumentalists who face MPA with different things at stake.

PA

“Everyone is human being. They are going to face it.”

PB

“A lot of performers do get nervous”

PC

“Tell them that what you’re feeling is normal”

PD

“Everyone will face it”

#### 5.4.3 It’s all about me now

Participants wished to bring their uniqueness into their performances. The core processes of Values and Committed Action were very stronly seen by the 5th and 6th sessions. PB accepted that it might mean choosing the right audience who will appreciate his music. Once values were clarified, there was no going back to simply pleasing the/any audience. Performances were now intrinsically motivated for one’s pleasure and having fun rather than to prove their worth to others and themselves. Because of this, participants looked for more opportunities to sing in situations outside of their comfort zones. They also looked forward to honing and nurturing their talents and saw It as a progressive journey. The participants believed that the audience and their peers would be influenced by the music they created.

#### 5.4.4 Change in symptom levels

At the beginning of the sessions, the participants were keenly aware of their symptoms, keeping track of when they began and how they changed or stayed the same during and after their singing. In the exit interviews, the participants reacted to their symptoms in various ways.

Two participants (B and D) noticed that their symptoms had reduced. One of them (D) noticed the usual beginning of the symptoms, but she learnt to defuse from the usual response to the trigger and consequently noticed that the symptoms reduced during performance.

PA could not tell what happened to his symptoms. Either he had not noticed because he had been focusing on task-related details, or his symptoms might have reduced. This participant had mainly cognitive symptoms. Hence, being mindful and staying present while performing could have shifted his focus off his obtrusive thoughts during singing. PD remarked that he did not notice the symptoms which had bothered him earlier. It is also unclear in this case whether the symptoms disappeared. It was clear that irrespective of whether the symptoms reduced, disappeared or stayed the same, the participants were no more concerned about getting rid of the symptoms. Instead, they chose to focus on their performance and be aware of what was happening in the present moment.

The pre-coaching questionnaire and interviews focused mostly on the symptoms and perceived nervous states before performance. However, the post-coaching interviews and questionnaires focused more on the learning and the future application of the learning. This was done on purpose to determine the change in the level of pre-occupation with symptoms.

#### 5.4.5 I want to tell my peers about this

While earlier, the participants had been shy about talking about their MPA to their peers, the coaching changed that aspect in several ways. All of them indicated that they were more open to talking about it because they no longer saw it as an extended imperfection of themselves as people. They also wished to spread this good news to fellow performers.

Participants (D & A) who saw mistakes as the worst thing that could happen during performances now welcomed them as a way of improving and learning. The bigger picture of artistic development was being taken into consideration. This helped them commit to regular practice schedules irrespective of whether there was a performance.

With the change in perception of self and self-conscious feelings of shame and guilt due to the development of the core processes of Cognitive Defusion and Self as Context, participants seemed willing to talk about their thoughts related to MPA. Participants also wanted these benefits for their peers and other performers as well. They could think of several people in their circle who would benefit from ACC. The participants also felt that ACC had helped them in other areas of life apart from singing. Along with accepting themselves and others as humans, participants also wished for other performers also to be able to do their best. PC, who had been the only one who said that his peers confided freely about their MPA to each other, felt that they should also know that help is available.

## 6 Discussion

The current study sought to examine whether Acceptance and Commitment Coaching (ACC) would be a feasible intervention that can be used by singing teachers to alleviate Music Performance Anxiety (MPA) faced by adolescent singers. Additionally, the study aimed to examine whether the use of ACC had the potential to help adolescent singers cope with inevitable development-related anxieties and keep them engaged in singing during their pubertal years. Based on the suggestion that flow states are a good antidote to MPA and for keeping an individual returning to an activity, the study used a positive psychology approach to coaching adolescents using the ACT framework (Csikszentmihalyi, 1975; Hill and Oliver, 2019).

Results showed evidence of an increase in psychological flexibility in the participants as well as the ability to defuse MPA-related thoughts and accept their MPA symptoms. This is consistent with results from a previous ACC study by a singing teacher on a university student (Shaw et al., 2020). The results are similar to ACT psychotherapy for MPA studies with seven vocal students (Juncos et al., 2017). It appears that the adolescent participants learnt to de-fuse from their MPA-related thoughts and accepted their MPA symptoms, behaving more flexibly in the presence of these symptoms, similar to the results seen in the previous studies on older vocal students. The results of this study are also in keeping with the previous study on ACC being an effective coaching framework that can be used by a singing teacher with no training or education in psychotherapy (Shaw et al., 2020). This change in behavior due to psychological flexibility was not seen immediately but took between 4 to 6 weeks. During this time the participants went through stages of confusion, boredom and restlessness, mainly at not being able to focus for a long time and also frustration at not being able to complete an exercise. However, they were motivated when they began to see changes in their ability to focus, thus helping them move on to the next step. They also realized that change takes time and hard work. The neuroscience of habit and subsequent behavioral changes is especially important for the coach to know, in order to be able to recognize the typical characteristics of the change process and to be sensitive to the feelings of confusion and impatience expressed by the participants (McKay and Kemp, 2018).

Findings revealed that among the biggest concerns of adolescent singers was the presence of an audience, both real and imaginary. This is in keeping with previous studies done on the effect of audience presence on performance (Slee, 1993; LeBlanc et al., 1997), the capacity for adolescents for increased abstract thinking (Steinberg, 2005) and the concern that other people are constantly judging and assessing them (Vartanian, 2000). Additionally, there was evidence of fear of failure, making mistakes and maladaptive perfectionistic tendencies that showed that achievement situations such as music performance were not viewed as opportunities for improvement but as threatening and potentially shameful. These findings align with previous studies in these areas (McGregor and Elliot, 2005). The participants also showed a lack of motivation to practice on a regular basis, which could be attributed to frustration in adjusting motor coordination of their rapidly growing bodies (McGregor and Elliot, 2005). Participants were also seen to avoid uncomfortable performance situations which further exacerbated their fears (McGregor and Elliot, 2005). The older participants also showed hints of shame at having MPA symptoms, which is in line with studies which show the increasing trajectory of shame along with the increased potential for abstract thinking (Orth et al., 2010; Mojallal et al., 2021).

After the completion of the ACC sessions, the participants reported not only psychological flexibility in the presence of MPA symptoms, and defusion from MPA-related thoughts in combination with values-based action, but they also began to normalize the making of mistakes during the performance, instead seeing them as ways to improve in their future performances. Mistakes and failure as singers were no longer seen as an extension of themselves but as a natural part of them. This could be a clue that ACC can be used to alleviate perfectionistic tendencies and fear of failure in adolescent singers. However, more longitudinal studies are needed in this regard to assess the long-term behavioral change in them. As dimensions of MPA shift over time in relation to performance context and development of the limbic system of the brain, it is also necessary to conduct studies to assess changes in adolescent MPA over an extended period of time (Patston and Osborne, 2015).

The participants also showed increased acceptance of negative feelings like nervousness and anxieties being part of them. This is in keeping with previous studies which show the importance of experiences in the development of self-identities and self-concepts, which are considered the key tasks in adolescence (Markus and Wulf, 1987; Crocetti et al., 2008). Since the audience for these participants comprised mainly of their schoolmates and their families, there seemed to be a higher risk involved with regard to public performances, which is the fear of judgment and exclusion by their peers, in addition to fears involved with performing for any audience in general. With regard to peers being the audience, the participants faced the potential risk of rejection and evaluation by their own friends who would be part of the audience. The three older participants also mentioned that MPA was not discussed freely within their peer groups. These findings are in keeping with studies which showed the following: adolescence is a critical time to form and maintain interpersonal relationships (Baumeister and Leary, 1995), the development of a social identity during adolescence (North and Hargreaves, 1999), high risk for social anxiety development during this period (Zimmer-Gembeck et al., 2021), the presence of a small peer group seen to increase MPA in high school music students (LeBlanc et al., 1997), young adolescents increased sensitivity to evaluation or criticism (Slee, 1993; Semper et al., 2016; Hoxter, 2017), development of maladaptive schemas due to experiences of shame (Mojallal et al., 2021), development of a negative self-concept during adolescence in response to shame experiences (Ogilvie, 1987), young people rarely seeking help for their phobias for fear of being ostracized (Dobos et al., 2019) and the heightened importance of peer group inclusion in middle school (Flanagan et al., 2008). By the end of the ACC sessions, the participants showed an increased acceptance of themselves and their peers and the idea of being human with flaws. This could be a potential benefit of using ACC on adolescent singers as it could help with the developmental need of forming a healthy social identity. Further studies are needed to investigate how psychologically flexible adolescents could impact their peer groups.

The older participants also mentioned experiencing flow-like states in their performances toward the end of the ACC sessions. While earlier they had trouble focusing on their song, they could now be in the moment and focus on various aspects like lyrics, expression and communicating the song because they had defused from MPA symptoms and focused on their values instead. This is in keeping with the study that it is possible for non-expert singers to experience flow (Barlow et al., 2004; Broomhead, 2010). Experiencing flow could motivate adolescents to keep coming back to the activity of singing owing to the release of dopamine while indulging in pleasurable and rewarding activities (Cohen and Bodner, 2019). It is unclear whether the younger participants experienced these flow states. It is possible that low levels or lack of increase in flow in the younger adolescents in this study were due to the absence of perceived skill/challenge balance in them, similar to studies conducted by Cohen and Bodner (2019) and Cohen and Bodner (2019). Overall, ACC has the potential to help adolescent singers cope with inevitable development-related anxieties.

### 6.1 Study limitations

This study is not without limitations. Given it was a qualitative study with four participants, direct conclusions about ACC’s effectiveness in helping adolescent MPA cannot be made. It is possible that the participants’ improvements related to psychological flexibility were due to a demand characteristic that positive change was expected, which threatens the validity of the study. However, the pattern of results is similar to those of the vocal students of Shaw et al. (2020), Juncos et al. (2017) and Juncos and Markman (2015). These trends across studies strengthen the possibility that the changes and psychological flexibility in the participants were due to ACC interventions. The study was also conducted over Zoom, disrupting the conversation flow with participants with poor internet connectivity. While all the participants felt this was a minor inconvenience in comparison to other benefits like being in the comfort of one’s home and being able to participate from other locations, it would still be useful to conduct a similar study using in-person sessions. It must also be noted that there was no attempt to record the changes in performance quality itself. It would be beneficial to include this aspect in future studies with adolescent singers.

### 6.2 Future recommendations

There is a need for more studies on using ACT with child and adolescent singers and ACC by singing teachers for this age group. This would help in customizing and refining the ACT principles for these age groups with special considerations based on their cognitive and physical development. The participants also indicated that they were willing to talk about their MPA after undergoing the sessions. In particular, they indicated they were keen to talk to their peers about it and help them. It would be interesting to look into the impact of peer influence by psychologically flexible adolescent singers.

Parents and singing teachers seemed to play an important part in the singer’s microsystem, potentially influencing the adolescent’s psychological flexibility and contributing to the development of their musical identity (Lamont, 2017). It has been suggested that the parents also undergo ACC so that they can not only help their child between sessions but also prevent any conflicting coping strategies being reinforced or advocated by them (Black, 2022). However, no study has been conducted to date on including parents in MPA coaching using ACC. I believe future research evaluating this aspect is much needed.

## Data availability statement

The original contributions presented in the study are included in the article/supplementary material, further inquiries can be directed to the corresponding author/s.

## Ethics statement

The studies involving humans were approved by Research Ethics committee of the University of Wales Trinity St David. The studies were conducted in accordance with the local legislation and institutional requirements. Written informed consent for participation in this study was provided by the participants’ legal guardians/next of kin. Written informed consent was obtained from the individual(s) for the publication of any potentially identifiable images or data included in this article.

## Author contributions

AP: Writing – original draft, Writing – review & editing. DJ: Supervision, Writing – review & editing. DW: Supervision, Writing – original draft.
